# Conduction System Pacing for Cardiac Resynchronization Therapy in Heart Failure with Reduced Ejection Fraction

**DOI:** 10.3390/jcm14030917

**Published:** 2025-01-30

**Authors:** Saurab Karki, Pallavi Lakra, Kaushik Kumar, Shiavax J. Rao

**Affiliations:** 1 Internal Medicine, MedStar Franklin Square Medical Center, Baltimore, MD 21237, USA; 2 Internal Medicine, Hartford HealthCare Medical Group, West Hartford, CT 06117, USA; 3 Pulmonary and Critical Care Medicine, Rutgers Robert Wood Johnson Medical School, New Brunswick, NJ 08901, USA; 4 Cardiovascular Medicine, University of Virginia, Charlottesville, VA 22908, USA

**Keywords:** cardiac resynchronization therapy, biventricular pacing, conduction system pacing, his-bundle pacing, left bundle branch area pacing, heart failure with reduced ejection fraction

## Abstract

Most patients with heart failure exhibit ventricular dyssynchrony, which is addressed by cardiac resynchronization therapy, traditionally through the use of biventricular pacing (BVP) devices. Despite this, around 30% of patients do not achieve the desired clinical outcome, and echocardiographic findings show that some patients deteriorate even further. Conduction system pacing (CSP) is a more physiologic pacing technique and includes his-bundle pacing (HBP) and left bundle branch area pacing (LBBAP). In this review, we further discuss and compare various CSP techniques for cardiac resynchronization therapy in patients with heart failure with reduced ejection fraction. After analyzing the current state of the literature on this topic until 2023, eight studies were included in this review and consisted of two trials and five observational studies with a total of 2841 patients. Both BVP and CSP resulted in improved outcomes in terms of NYHA class, QRS duration, and left ventricular ejection fraction over time. These effects were more pronounced in patients undergoing CSP, as the technique is more physiological and results in the synchronized activation of the ventricles. LBBAP yielded better outcomes compared to BVP and resulted in fewer heart failure hospitalizations and a lower all-cause mortality rate.

## 1. Introduction

Heart failure (HF) is a constellation of complex clinical features as a consequence of anatomical or physiological disturbances leading to the inability to meet the circulatory demands of the body [1]. The clinical features are further supported by elevated natriuretic peptides with or without the presence of systemic or pulmonary vascular congestion [2]. Heart failure can be broadly classified based on the left ventricular ejection fraction (EF) as reduced (HFrEF) when EF is ≤40%, preserved (HFpEF) when the EF is ≥50%, and mid-range or mildly reduced (HFmrEF) when the EF is 41–49% [3]. This classification system has clinical significance in terms of patient management [4]. Heart failure with improved ejection fraction (HFimpEF) is defined as an initial baseline EF of ≤40%, with a ≥10-point improvement in EF from baseline, and with an EF >40% when measured for the second time [2,3]. With approximately 64.3 million people affected by heart failure worldwide, there is a huge burden imposed on healthcare expenses [5]. The annual median cost for total medical services related to its care is around USD 24,383 per patient [6]. If the prevalence continues to increase, the cost of care will increase significantly within the United States (US) to USD 244 annually by 2030 for approximately 10 million HF patients [7].

Most patients with heart failure exhibit ventricular dyssynchrony mechanically or electrocardiographically, which can be the therapeutic target using multisite pacing [8,9]. Cardiac resynchronization therapy (CRT) is a specialized treatment, involving pacing of both the right and left ventricles to maximize ventricular synchrony [10]. Also known as biventricular pacing, this is a safe and effective treatment alternative for medical therapy refractory HF cases, indicated in advanced disease by an EF ≤35%, significant cardiac symptoms, particularly New York Heart Association (NYHA) classes of III and IV, and conduction system disorder [11]. Despite this, around 30% of patients do not achieve the desired clinical outcome, and echocardiographic findings show that some patients deteriorate further following conventional pacing techniques [12]. Conduction system pacing (CSP) is a more physiologic pacing technique and includes his-bundle pacing (HBP) and left ventricular bundle branch area pacing (LBBAP) [13]. In this scoping review, we further discuss and compare various CSP techniques for cardiac resynchronization therapy in patients with HFrEF.

## 2. Materials and Methods

### 2.1. Search Strategy

To identify and retrieve the relevant articles up to 2023, a comprehensive search of the PubMed database was conducted. A combination of Medical Subject Heading (MeSH) terms and search words related to the topic of interest were used. MeSH and search terms included “cardiac resynchronization therapy”, “conduction system pacing”, “biventricular pacing”, “his-bundle pacing”, “left bundle branch pacing”, and “heart failure with reduced ejection fraction” along with appropriate Boolean operators. The reference lists of the reviewed articles were screened as well.

### 2.2. Eligibility Criteria

Inclusion criteria for the study included completely available randomized or nonrandomized controlled trials (RCTs) and observational studies comparing HBP or LBBAP with BVP in patients with HFrEF. We excluded reviews, meta-analyses, animal studies, protocol opinions, studies considering HFpEF, or those where population, intervention, and outcomes were not relevant or primary data were not available.

### 2.3. Data Extraction

All appropriate articles were reviewed. Extracted data included baseline patient demographics, study type, outcomes in terms of clinical (NYHA class), electrophysiological (QRS duration) and mechanical (LVEF) parameters, and complications.

### 2.4. Included Studies

**Figure 1:**
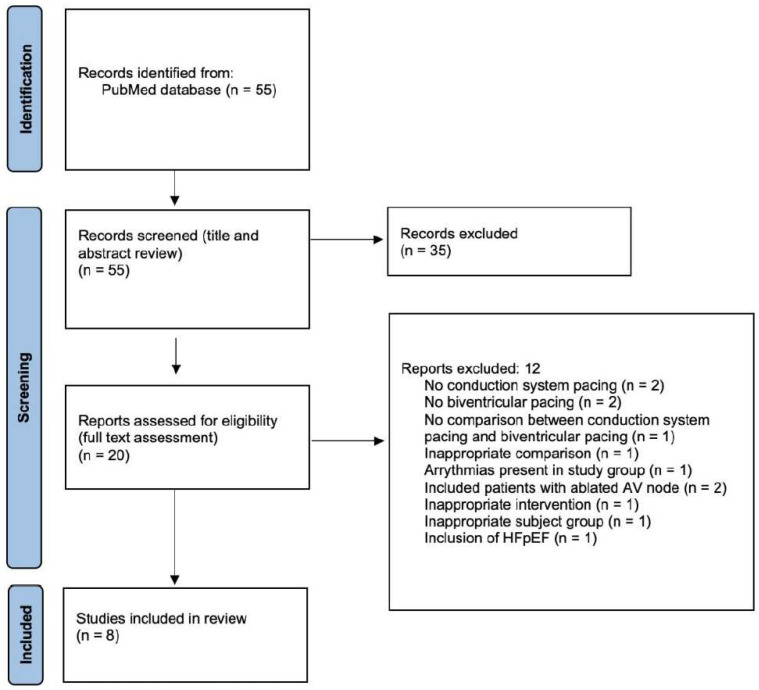
PRISMA flow diagram outlining the identification, screening, and inclusion of studies.

The electronic database search yielded 55 articles. The titles and abstracts of these articles were screened and 35 articles were excluded as they did not pertain to the topic of interest. Twenty articles were included in full-text analysis and 12 were excluded. Subsequently, 8 studies were included for review. A flow diagram outlining the search strategy, screening, and data extraction is highlighted in Figure 1.

## 3. Results

The eight studies included in this review consisted of two trials and five observational studies with a total of 2841 patients. All studies included HFrEF patients who underwent either BVP or CSP with either HBP or LBBAP as part of their treatment. Four studies compared LBBAP with BVP, three studies compared CSP with BVP, and one study compared HBP with BVP. Three studies assessed the clinical grade using the NYHA functional class, five studies assessed electrocardiographic findings using the change in QRS duration, and seven studies compared the mechanical changes using the change in LVEF on echocardiography. Two studies discussed the success rate of the procedure being performed while five studies discussed the complications that developed following the procedures. The key results are summarized in Table 1.

### 3.1. Change in NYHA Functional Class from Baseline

Three studies (one RCT, two observational studies) investigated the impact of pacing strategy on the change in the NYHA functional class from baseline. Chen et al. [14] showed that LBBAP resulted in significantly greater improvement in the NYHA class at 6 months compared to BVP (12.24% had class III-IV in the LBBAP group vs. 25.49% in the BVP group) from a baseline patient population, where 91.84% from the LBBAP group and 88.24% from the BVP group had NYHA class III-IV as shown in Table 1. At 1-year follow-up, 4.08% of patients from the LBBAP group and 19.61% of patients from the BVP group had NYHA class III-IV symptoms. Likewise, another study by Liu et al. [15] also showed a significantly greater improvement in the NYHA functional class in the LBBAP group compared to the BVP group (−1.6 ± 0.6 vs. −0.9 ± 0.8, respectively; *p* = 0.001) from baseline over a short-term follow-up period (mean 4.0 ± 1.4 months; range 3 to 6 months). However, a recent RCT by Wang et al. [16] did not show a statistically significant difference in the change in functional class between LBBAP and BVP groups at 6 months (−1.22 ± 0.11 vs. −1.10 ± 0.11).

### 3.2. Change in QRS Duration

Five included studies (two RCTs, three observational studies) investigated the impact of CSP on the change in QRS duration (QRSd) over time compared to baseline. Two studies compared LBBAP with BVP, one compared HBP with BVP, and two compared CSP (without further categorization as HBP or LBBAP) with BVP. Wang et al. [16] performed a per-protocol analysis to compare the change in QRSd between LBBAP and BVP groups and it was found to be shortened from 174.6 ± 14.3 ms to 129.2 ± 10.8 ms (LBBAP group) and from 174.7 ± 14.1 ms to 138.5 ± 10.6 ms (BVP group). Similarly, Liu et al. [15] also reported a significantly shortened QRSd for the LBBAP group vs. the BVP group (ΔQRSd −64.1 ± 18.9 ms vs. −32.5 ± 22.3 ms, respectively, *p* < 0.001). Tan et al. [17] compared CSP (without further characterization as LBBAP or HBP) to BVP. Their study results demonstrated a reduction in QRSd from a pre-implant status of 156 ± 26 ms vs. 158 ± 26 ms (*p* = 0.99) to a post-implant status of 149 ± 16 ms vs. 127 ± 28 ms (*p* < 0.001) between the BVP and CSP groups, respectively. Ezzedine et al. [18] also compared CSP to BVP and the study showed QRSd shortening from 150.6 ± 32.9 ms to 128 ± 26.5 ms (*p* < 0.001) among the CSP group compared to the BVP group, where there was no significant change in QRSd (150.9 ± 33.3 to 157.3 ± 44; *p* = 0.152). The paced QRS duration was 128 ± 26.5 ms vs. 157.3 ± 44 ms (*p* < 0.001) among the two groups, respectively, which showed statistically significant narrowing in the CSP group compared to the BVP group. Finally, Højgaard et al. [19] showed a shortening of QRSd from 168 ± 15 ms to 134 ± 19 ms (*p* ≤ 0.001) in the HBP group compared to that of the BVP group, where QRSd shortening was from 163 ± 14 ms to 129 ± 14 ms (*p* < 0.001).

### 3.3. Change in LVEF from Baseline

Seven studies discussed the impact of the type of CRT on the change in LVEF, with four studies comparing LBBAP to BVP. Wang et al. [16] demonstrated a significantly higher LVEF at 6 months for the LBBAP group compared to the BVP group 47.58 ± 12.02% vs. 41.24 ± 10.56% (*p* = 0.008). Both groups had similar LVEF at baseline 29.05 ± 5.09% vs. 28.36 ± 5.30%, respectively (*p* = 0.522). They also reported a change in LVEF of 21.08 ± 1.91% vs. 15.62 ± 1.94% (*p* = 0.039) over 6 months in the LBBAP and BVP groups, respectively. Chen et al. [14] demonstrated a change in LVEF from 29.05 ± 5.09% vs. 28.36 ± 5.30% (*p* = 0.522) at baseline to 47.58 ± 12.02% vs. 41.24 ± 10.56% (*p* = 0.008) at 6-month follow-up for the LBBAP and BVP groups, respectively. Vijayaraman et al. [20] found that LVEF improved from 27 ± 6% to 41 ± 13% (*p* < 0.001) with LBBAP compared to an increase from 27 ± 7% to 37 ± 12% (*p* < 0.001) with BVP, with significantly greater change from baseline with LBBAP (13 ± 12% vs. 10 ± 12%; *p* < 0.001). Liu et al. [15] defined the echocardiographic response rate as an absolute increase of ≥10% in LVEF compared to baseline. Accordingly, the response rate favored the LBBAP group compared to the BVP group (88.9% vs. 68.6%). Likewise, the non-response rate also favored the LBBAP group compared to the BVP group (11.1% vs. 31.4%), respectively.

Three studies compared the LVEF between CSP and BVP. Another study by Vijayaraman et al. [21] showed that LVEF improved in both groups during a mean follow-up of 27 ± 12 months and was greater after CSP compared to BVP (39.7 ± 13% vs. 33.1 ± 12%; *p* < 0.001). Ezzeddine et al. [18] found that LVEF improved from 33.2 ± 9.5% to 43.6 ± 11.5% (*p* < 0.001) in the CSP group compared to an improvement from 35.3 ± 12% to 42.7 ± 2.8% (*p* < 0.001) in the BVP group, with a mean change of (10.4 ± 10.9% vs. 7.3 ± 9.4%; *p* = 0.037) between the groups, respectively. Likewise, the proportion increase in LVEF by ≥5% was 74% among the CSP group compared to 60% with the BVP group. In the prospective cohort study by Tan et al. [17], the baseline EF of the BVP and CSP groups was 28 ± 7% and 29 ± 9% (*p* = 0.59), respectively, which, over a period of 6 months, improved to 32 ± 11% and 39 ± 13% (*p* = 0.02), respectively. The changes in LVEF in the two groups were 3 ± 10% vs. 12 ± 14%, (*p* < 0.01), respectively, showing more improvement in the CSP group.

### 3.4. Complications and Outcomes

Five of the included studies addressed complications and outcomes. Vijayaraman et al. [21] reported increased mortality among the BVP group compared to the CSP group (24% vs. 17%), although the difference was not statistically significant (*p* = 0.09). However, this study showed a significantly higher rate of heart failure hospitalization (HFH) among patients in the BVP group (34%) compared to the CSP group (15%). Tan et al. [17] also reported a significantly higher rate of HFH in the BVP group compared to the CSP group (38% vs. 19%, respectively, *p* = 0.04). All-cause mortality was significantly lower in the CSP group compared to the BVP group (10% vs. 40%, *p* = 0.001). Ezzeddine et al. (17), demonstrated no significant difference in HFH between the CSP and BVP groups (14% vs. 9%, respectively, *p* = 0.248). Furthermore, in their study, the mortality rate between the two groups demonstrated no significant difference (15% vs. 20%; *p* = 0.314). Chen et al. reported that HFH was 2 in the LBBAP group while it was 5 in the BVP group at the end of 1 year (*p* = 0.437). No mortality was recorded during the study period. A study by Vijayaraman et al. (19) compared the mortality rate between BVP and LBBAP groups and found it to be 17% and 12%, respectively, with a *p*-value of 0.006. The HFH rate was 19% in BVP and 12% in LBBAP, with a *p*-value of less than 0.001.

## 4. Discussion

Our review includes eight studies, consisting of two trials and five observational studies. The main findings of the review include an improvement in the NYHA class, a shortening of QRSd, and an improvement in LVEF for both CSP and BVP, although the effects are more pronounced with CSP. Additionally, HFH and mortality are also notably higher with BVP compared to CSP. CRT using LBBAP also achieved better outcomes compared to the BVP group along with fewer complications.

Improvement in the NYHA functional class can be a clinical guide to assess the efficacy of treatment. Three studies showed that the use of BVP and LBBAP can improve the NYHA functional class, with two of them showing a statistically significant improvement in the LBBAP group compared to BVP. The findings are supported by a meta-analysis by Gin et al., with 4 RCTs and 11 observational studies with 1211 patients. The analysis shows a statistically significant improvement in the NYHA functional class with the use of CSP over BVP. In the sub-group analysis comparing LBBAP and BVP, there was a statistically significant improvement in the NYHA functional class, more so with LBBAP than with BVP [22]. Another meta-analysis included four non-randomized controlled trials and showed a significantly improved NYHA functional class with LBBAP compared to BVP [23]. Likewise, a meta-analysis by Jin et al., which included nine observational studies and one RCT with 616 patients, also showed a greater improvement in the NYHA functional class with the LBBAP group over the BVB group [24]. Patients with NYHA functional class III or IV are less likely to tolerate medical therapy due to reasons such as hyperkalemia, hypotension, or renal dysfunction, which develops with higher doses of beta blockers or ACE inhibitors. With CRT, the effects of medical therapy can be maximized and sometimes medications can be reintroduced among patients who were previously intolerant, leading to an improvement in the NYHA functional class [25]. A possibility for better outcomes in LBBAP compared to BVP could be due to the correction of complete LBBB present with HF, which can restore electromechanical synchrony of the left ventricle, ultimately improving the clinical parameter [26].

QRSd can be used as a guide to predict electrical synchronization and assess the efficacy of treatment [27]. From our review, four studies showed a reduction in QRSd following the use of BVP while one study did not. Additionally, two studies showed a shortening of QRSd with CSP, two studies demonstrated QRSd shortening with LBBA, and one study showed QRSd shortening with HBP. In their meta-analysis, Gin et al. found that the use of CSP led to a reduction in QRSd in both CSP and BVP groups. The sub-group analysis demonstrated a significant reduction in QRSd with both LBBAP and HBP compared to BVP [22]. Likewise, the meta-analyses by Liu et al. and Jin et al. also demonstrated a significant reduction in QRSd with the LBBAP group compared with the BVP group [23,24]. With the use of BVP, the stimulation is initiated from the right ventricle endocardium and left ventricle epicardium and spreads down the myocardial tissue. Similarly, HBP achieves synchronization from proximal LBB while LBBAP captures the distal LBB, and the wavefront is spread more physiologically in the conduction system compared to BVP [28,29]. This is a potential explanation for the achievement of significant QRSd shortening to a greater extent with CSP than with BVP.

Improvement in LVEF is an important therapeutic target and can also be used as a guide to assess the effectiveness of treatment while managing HF patients. In our review, seven studies showed improvement in LVEF with the use of either BVP or CSP. In comparing between the groups, the studies showed a statistically significant improvement with LBBAP over BVP, and three studies showed a statistically significant improvement with CSP in general over BVP. One study mentioned the non-response rate, which was higher with BVP compared to CSP while another study mentioned the hyper-response rate was significantly higher with LBBAP compared to BVP. Based on the meta-analysis by Gin et al., the use of CSP resulted in a significant increase in LVEF compared to BVP, with sub-group analysis revealing a significant improvement in LVEF with LBBAP [22]. Similarly, other meta-analyses have also demonstrated significant improvement in LVEF in HFrEF with LBBAP compared to BVP [23,24]. Given the various conduction abnormalities (atrioventricular, interventricular, or intraventricular) associated with chronic HF, the use of BVP synchronizes the activation of both ventricles and appears to improve hemodynamics. This would be attained by increasing the filling time of the left ventricle, decreasing any associated mitral regurgitation, and reducing the septal dyskinesis due to delayed LV activation and contraction [30]. LBBAP works via the activation of the left bundle branch area, resulting in electrical stimulation traveling through the intrinsic conduction system rather than through intercellular conduction. This effect may help HF patients maintain left ventricular systolic synchronization more effectively. LBBAP also tends to improve myocardial work efficiency (MWE) throughout all left ventricular segments with the exception of the lateral segment while BVP was not as effective as LBBAP at increasing MWE and minimizing the wasted work in the anterior and posterior segments [15].

We also reviewed the outcomes and complications, particularly all-cause mortality and HFH with different pacing techniques. Among three studies comparing BVP and CSP groups, two studies showed a statistically significant increase in the HFH rate and one study showed a statistically significant increased mortality rate with BVP compared to CSP techniques. One study showed significantly increased HFH in BVP over LBBAP, while another study showed no statistically significant difference between the groups. Regarding mortality, one study had no recorded mortality while another study revealed no significant difference in mortality between the LBBAP and BVP groups. In the meta-analysis by Liu et al., no statistically significant differences in complications were present between LBBAP and BVP groups, and there was no mortality during the follow-up time [23]. Another meta-analysis by Jin et al. demonstrated a statistically significant reduction in HFH with LBBAP versus BVP, while there was a comparably low mortality rate between both groups [24].

It is important to note that CRT may benefit both patients with ischemic cardiomyopathy (ICM) and dilated non-ischemic cardiomyopathy (DCM). A 2019 study enrolling 88 patients with ICM (59% of the total sample) or DCM (41% of the total sample) undergoing traditional CRT with a mean follow-up period of 76.4 months found that ICM patients showed increased mortality compared to DCM patients [31]. Studying the longitudinal application of CSP in large samples of patients stratified as ICM or DCM will be an important future direction.

There are some limitations in our review study, starting with the low number of included studies given the paucity of primary data on outcomes related to these emerging pacing techniques. Furthermore, there was a relatively low number of RCTs, and observational studies are prone to selection and reporting bias. Each individual included study has their own limitations. There was a paucity of data on HBP compared to LBBAP, limiting the comparison between the two sub-groups of CSP. Larger cohort studies and RCTs with greater sample sizes are warranted to address this gap. Furthermore, several of the included studies had small sample sizes, particularly those focusing on HBP, effectively making it challenging to draw definitive conclusions regarding the relative efficacy of different CSP techniques. Lastly, it is important to note that the included studies in this scoping review differed in design, duration, and outcome measures, complicating direct comparisons between CSP and BVP. Future studies with larger sample sizes, larger data sets, and similar outcome endpoints would allow for more direct comparisons via meta-analyses or time-based analyses.

Nevertheless, based on our review, both CSP and BVP techniques result in improved outcomes in terms of the NYHA class, QRSd, and LVEF. The effects have been shown to be more pronounced with the CSP technique, likely due to it being more physiological and resulting in the synchronized activation of the ventricles. LBBAP tends to have better outcomes compared to BVP, such as lower rates of HFH and lower or comparable all-cause mortality.

## Figures and Tables

**Table 1:** Key findings and outcomes of included studies.

Study	Type	Study Sample	NYHA Functional Class	QRS Duration	LVEF	Outcomes
Chen et al. (2022) [14]	Non-randomized, prospective, multi-center, observational	49 LBBAP vs. 51 BVP	% of patients with NYHA Class III–IV: Baseline: LBBAP 91.84% vs. BVP 88.24% (*p* = 0.741) 6 months: LBBAP 12.24% vs. BVP 25.49% (*p* = 0.126) 1 year: LBBAP 4.08% vs. BVP 19.61% (*p* = 0.028)	Not assessed	Baseline: LBBAP 29.05 ± 5.09% vs. BVP 28.36 ± 5.30% (*p* = 0.522) 6 months: LBBAP 47.58 ± 12.02% vs. BVP 41.24 ± 10.56% (*p* = 0.008) 1 year: LBBAP 49.10 ± 10.43% vs. BVP 43.62 ± 11.33% (*p* = 0.021)	HFH at 6 months: LBBAP 2.04% vs. BVP 5.88% (*p* = 0.618) HFH at 1 year: LBBAP 4.08% vs. BVP 9.8% (*p* = 0.437) No mortality during study period
Liu et al. (2021) [15]	Multi-center, prospective cohort	35 BVP vs. 27 LBBAP	Improvement in overall NYHA class: LBBAP −1.6 ± 0.6 vs. BVP −0.9 ± 0.8 (*p* = 0.001)	Significantly QRSd narrowing: ΔQRSd LBBAP −64.1 ± 18.9 vs. ΔQRSd BVP −32.5 ± 22.3 ms (*p* < 0.001)	Echocardiographic response rate (≥10% absolute increase in LVEF compared to baseline) LBBAP 88.9% vs. BVP 68.6% No-response rate: (11.1% vs. 31.4%)	Not assessed
Wang et al. (2022) [16]	RCT	20 LBBAP vs. 20 BVP	6-month ΔNYHA class: LBBAP −1.22 ± 0.11 vs. BVP −1.10 ± 0.11 (*p* > 0.05)	ΔQRSd for LBBAP from 174.6 ± 14.3 ms to 129.2 ± 10.8 ms (*p* < 0.05) ΔQRSd for BVP from 174.7 ± 14.1 ms to 138.5 ± 10.6 ms (*p* < 0.05)	6-month ΔLVEF: LBBAP 21.08 ± 1.91% vs. BVP 15.62 ± 1.94% (*p* = 0.039)	Not assessed
Tan et al. (2023) [17]	Registry-based, prospective cohort	48 CSP vs. 48 BVP	Not assessed	Pre-implant QRSd: BVP 156 ± 26 ms vs. CSP 158 ± 26 ms (*p* = 0.99) Post-implant QRSd: BVP 149 ± 16 ms vs. CSP 127 ± 28 ms (*p* < 0.001)	Baseline LVEF: BVP 28 ± 7% vs. CSP 29 ± 9% (*p* = 0.59) 6-month LVEF: BVP 32 ± 11% vs. CSP 39 ± 13% (*p* = 0.02) ΔLVEF: BVP 3 ± 10% vs. CSP 12 ± 14% (*p* < 0.01)	HFH: BVP 38% vs. CSP 19% (*p* = 0.04) All-cause mortality: BVP 40% vs. CSP 10% (*p* = 0.001)
Ezzeddine et al. (2023) [18]	Multi-center, retrospective cohort	119 CSP vs. 119 BVP	Not assessed	ΔQRSd for CSP: from 150.6 ± 32.9 ms to 128 ± 26.5 ms (*p* < 0.001) ΔQRSd for BVP: from 150.9 ± 33.3 ms to 157.3 ± 44 ms (*p* < 0.152) Paced QRSd: CSP 128 ± 26.5 ms vs. BVP 157.3 ± 44 ms (*p* < 0.001)	ΔLVEF for CSP: from 33.2 ± 9.5% to 43.6 ± 11.5% (*p* < 0.001) ΔLVEF for BVP: from 35.3 ± 12% to 42.7 ± 2.8% (*p* < 0.001) Mean change in LVEF: CSP 10.4 ± 10.9% vs. BVP 7.3 ± 9.4% (*p* = 0.037)	HFH: CSP 14% vs. BVP 9% (*p* = 0.248) Unadjusted mortality: CSP 15% vs. BVP 20% (*p* = 0.314)
Højgaard et al. (2024) [19]	Double-blinded randomized pilot study	19 HBP vs. 31 BVP	Not assessed	ΔQRSd for HBP: from 168 ± 15 ms to 134 ± 19 ms (*p* ≤ 0.001) ΔQRSd for BVP: from 163 ± 14 ms to 129 ± 14 ms (*p* < 0.001)	Not assessed	Not assessed
Vijayaraman et al. (2023) [20]	Retrospective case–control	797 LBBAP vs. 981 BVP	Not assessed	Baseline QRSd: LBBAP 161 ± 28 ms vs. BVP 160 ± 25 ms (*p* = 0.63) Paced QRSd: LBBAP 128 ± 19 ms vs. BVP 144 ± 23 ms (*p* < 0.001)	ΔLVEF for LBBAP: from 27 ± 6% to 41 ± 13% (*p* < 0.001) ΔLVEF for BVP: 27 ± 7% to 37 ± 12% (*p* < 0.001)	HFH: LBBAP 12% vs. BVP 19% (*p* < 0.001) Mortality: LBBAP 12% vs. BVP 17% (*p* = 0.006)
Vijayaraman et al. (2022) [21]	Multi-center, retrospective, observational	258 CSP vs. 219 BVP	Not assessed	Baseline QRSd: CSP 150.5 ± 30 ms vs. BVP 160.7 ± 23 ms (*p* = 0.001) Paced QRSd: CSP 133 ± 21 ms vs. BVP 152 ± 24 ms (*p* < 0.001)	Baseline LVEF: CSP 26.4 ± 6.5% vs. BVP 26.1 ± 6.3% (*p* = 0.45) Paced LVEF (after 27 ± 12 months): CSP 39.7 ± 13% vs. BVP 33.1 ± 12% (*p* < 0.001)	Mortality or HFH: CSP 28.3% vs. BVP 38.4% (*p* = 0.013)

Abbreviations—BVP: Biventricular Pacing; CSP: Conduction System Pacing; HBP: His-Bundle Pacing; HFH: Heart Failure Hospitalization; LBBAP: Left Bundle Branch Area Pacing; LVEF: Left Ventricular Ejection Fraction; RCT: Randomized Controlled Trial.

## Data Availability

No new data were created or analyzed in this study. Data sharing is not applicable to this article.
